# Development of microsatellite markers and estimation of inbreeding frequency in the parasitoid wasp *Melittobia*

**DOI:** 10.1038/srep39879

**Published:** 2017-01-11

**Authors:** Jun Abe, Bart A. Pannebakker

**Affiliations:** 1 Faculty of Liberal Arts, Meijigakuin University, Yokohama, Kanagawa, Japan; 2 Research Institute for Integrated Science, Kanagawa University, Hiratsuka, Kanagawa, Japan; 3 Laboratory of Genetics, Wageningen University, Wageningen, The Netherlands

## Abstract

The parasitoid wasp *Melittobia* is an important insect for basic and applied biology. Specifically, their extremely female-biased sex ratios, which contrast to the prediction of pre-existing theories, are needed to be explained from the aspect of evolutionary biology. In this study, using next-generation sequencing, 20 microsatellite loci were developed and characterized for *M. australica*. The developed loci were used to analyze two populations, one from a mainland Japan and one from the Okinawa island region. Both populations showed a smaller observed heterozygosity than expected, and a high inbreeding coefficient. Deviations from Hardy-Weinberg equilibrium were recorded for the majority of the 20 loci, suggesting that both the populations are subdivided due to inbreeding as is expected by the reproductive biology in *Melittobia*. The sib-mating frequency in the two populations was calculated as 0.873 and 0.996, which is higher than the values estimated by the number of females observed in a host cocoon or cell, implying that closely related females lay eggs on a host. The microsatellite loci developed in this study can be used for more comprehensive analyses to identify genetic structure in natural populations for understanding their sex allocation behavior and for more generally evolutionary and population genetic studies.

Parasitoid wasps are important insects for both basic and applied biology^1^,^2^. Parasitoid females lay their eggs in or on the bodies of other insects (hosts). The developing parasitoid larvae consume the host tissues, resulting in the death of the host. The genus of *Melittobia* (Hymenoptera: Eulophidae) is a gregarious ectoparasitoid that is widespread in the whole world and attacks the larvae and pupae of a range of solitary wasps and bees, including mud dauber and mason wasps, and leaf cutting and mason bees^3^,^4^. Because their hosts include species commercially important for pollination management, such as *Osmia* and *Megachile* bees^5^,^6^,^7^, honey bees^4^,^8^, and bumblebees, in which the parasitoid invades mass-rearing factories and cause immense damage on the colonies^6^,^7^,^9^,^10^, *Melittobia* is feared as one of the most destructive natural enemies and is capable of causing enormous economic losses. At the same time, *Melittobia* exhibits a number of interesting traits that attract evolutionary biologists^3^,^4^, which include courtship behavior^11^,^12^, sex allocation^13^,^14^,^15^,^16^, lethal combat among males^17^,^18^,^19^, female dispersal dimorphism^20^,^21^, female cooperative dispersal^22^ and a mating system in which mothers mate with their sons^23^.

Specifically, the extremely female-biased sex ratios shown in *Melittobia* species are problematic, because they cannot be explained by the sex allocation theory^15^. Mating in *Melittobia* takes place within their natal patches (i.e. the breeding cells or cocoons of their hymenopteran hosts) before females disperse for a new host, after which males end their life without dispersal. Local Mate Competition (LMC) theory^24^ predicts that such a situation of inbreeding within patches favors a female-biased sex ratio, because it reduces competition for mates among sons and increases the number of potential mates for sons^25^. The frequency of inbreeding between sons and daughters is expected to decrease when increasing numbers of females (foundresses) lay eggs in a patch, and the female-biased sex ratios are predicted to approach to 50% sex ratio with increasing foundress numbers. However, in contrast to these predictions of LMC theory, which are supported by observations in many other parasitoid wasps^26^, the extremely female-biased sex ratios of *Melittobia* (about 1–5% males) change little in response to the number of foundresses on a host^13^,^15^,^27^,^28^,^29^.

Because of their minute size, and their concealed life style, highly polymorphic molecular markers, such as microsatellite markers are needed to better understand the life-history and behavior of *Melittobia* in the field. Although some molecular techniques and markers were applied for *Melittobia*^12^,^30^, only a single microsatellite marker is available in this genus until now^17^, which was used for behavioral studies in the laboratory^14^,^15^,^17^,^23^,^29^. The identification of an extensive panel of microsatellite loci with more polymorphism will facilitate investigations of the population structure and genetic relationship between wasps in natural field populations. In this study, we develop microsatellite markers using a next-generation sequencing approach (Ion Torrent PGM) and, using the developed markers, estimate the frequency of inbreeding in natural populations, which is a key population parameter to understand the evolution of sex allocation in *Melittobia*.

## Results and Discussion

### Sequence results and microsatellite content

By sequencing with Ion Torrent PGM, a total of 867 Mb sequence was obtained for *M. australica* with 3,547,911 reads, with the mean read length of 244 bp (raw sequence data deposited at NCBI Sequence Read Archive, accession number SRP091702). In total, 2,578,063 unique reads (72.7%) had a read length of over 150 bp, of which 90,261 reads contained one or more microsatellite loci (3.50%), resulting in a total of 95,305 microsatellite loci (Supplementary Table 1). In terms of microsatellite composition, dinucleotide motifs are the most abundant microsatellites in *M. australica*, making up to 86.8% of all microsatellites (Table 1). This is in agreement with reports on the microsatellite composition of other Hymenoptera, which also show an overrepresentation of dinucleotides compared to other insect groups^31^. Among total of 90,261 reads, primer pairs were designed for 32,180 microsatellite loci (35.7%, Supplementary Table 2). Of these, 67 loci (for 28 of di-, 31 of tri-, and 8 of tetranucleotide repeats) were selected for PCR amplification and investigation of polymorphism. In total, 41 loci were successfully amplified and 33 loci showed polymorphism in the Hiratsuka field population. Of these polymorphic loci, 20 loci that showed more than 2 alleles, and of which the rarest allele was observed in more than one female individual, were used for population genetic analysis below (Table 2; the sequence data of the loci were deposited at European Nucleotide Archive, http://www.ebi.ac.uk/ena/data/view/LT598812-LT598835).

### Population genetic analysis

Significant linkage disequilibrium (LD) was detected in the combined data from the Hiratsuka and Yaeyama populations for 6 pairs of loci in the both sexes (Mau15–Mau29, Mau15–Mau 41, Mau15–Mau60, Mau29–Mau41, Mau29–Mau60, Mau41–Mau60, Mau44–Mau45, and Mau57–Mau71), and 4 pairs only in the males (Mau37–Mau39, Mau37–Mau42, Mau37–Mau71, and Mau39–Mau42; Supplementary Table 3). Such patterns of LD may result from genetic linkage between the two loci existing adjacently on the same chromosome and/or from a subdivided population structure due to inbreeding. Although we cannot exclude genetic linkage between these loci, the biology of *Melittobia* does result in a structured population, which is the likely cause of LD in this species (see also below).

In the 20 microsatellite loci tested, the observed heterozygosity *H*_O_ ranged from 0 to 0.417 and the expected heterozygosity *H*_E_ from 0.304 to 0.743 in the Hiratsuka population (Table 2). Inbreeding coefficient *F* ranged from 0.353 to 1 among the examined loci (Table 2) and the value calculated over all the loci was 0.632. Significant deviations from HWE were detected with 12 out of 20 loci (Table 2). These deviations could be caused by the presence of null alleles for those loci^32^. However, because the 20 loci were amplified in all 12 haploid males used for polymorphism check in the present study and an additional 52 haploid males examined for an ongoing population genetic analysis in this population (J.A. unpublished data), the excess proportion of observed homozygotes in diploid females is not likely to be caused by the occurrence of non-amplifying null alleles due to nucleotide divergence in primer annealing regions. Null alleles could also be caused by differential amplification between the two alleles of heterozygotes^33^. Our data shows two suspicious alleles, 162 bp allele of Mau37 and 210 bp allele of Mau71, which were only amplified in the same single male. However, re-inspection of the fluorescence profiles of these loci failed to show vestiges of these suspicious alleles in any of the tested females, suggesting that these alleles are unlikely to be null alleles. Furthermore, removing the two loci from our data set resulted in a similar estimate of inbreeding (*F* = 0.613) in *M. australica* in the Hiratsuka population.

In the Yaeyama population, the 20 microsatellite loci were amplified in all 18 individuals examined. In the nine females, the observed heterozygosity *H*_O_ was very low (only one allele was heterozygous among the 20 loci), resulting in significant deviations from HWE in 19 out of 20 loci (Table 3). The inbreeding coefficient *F* equals unity in all loci except for Mau71 (Table 3), resulting in a high inbreeding coefficient over all the loci of 0.986. Because all the 20 loci were amplified in the nine haploid males tested, and no alleles were only detected in haploid males, the existence of null alleles at these 20 loci is highly unlikely in the Yaeyama population. Between the Hiratsuka and Yaeyama populations, a strong genetic differentiation was observed (*F*_*ST*_ = 0.401, ranging from 0.157 to 0.702, Table 3). This is further reflected in the high frequency of private alleles in both populations, of the 20 loci, 0.87 of the alleles was private to the Hiratsuka population and 0.70 to the Yaeyama population.

### Estimation of sib-mating frequency and foundress number

Excluding null alleles as the cause of the deviations from HWE observed in both the Hiratsuka and Yaeyama populations, a more plausible explanation is a locally subdivided population structure, as is to be expected based on the frequent inbreeding within local patches in *Melittobia*^3^,^4^. Using the estimated inbreeding coefficient over all the loci, the frequency of sib-mating was calculated as *α* = 0.873 and 0.996, and the number of ovipositing females on a patch as *n* = 1.15 and 1.00 for the Hiratsuka and Yaeyama populations, respectively. However, the estimated value for female number may be underestimated (and that for sib-mating frequency may be overestimated), since the harmonic mean number of females existing in a host cocoon in the Hiratsuka field population was 2.29 (the arithmetic mean number of the females ± standard deviation was 9.47 ± 11.9; J.A. unpublished data), although similar data was not available for the Yaeyama population. Possible explanations for the lower estimate of female number based on microsatellites include that (1) the females in the same host cocoon do not contribute equal numbers of offspring, and/or (2) the females ovipositing in the same host cocoon are relatives. Both possibilities are likely in the case of *Melittobia*. First, females can use two ways to access the appropriate stage of hosts: they can either sneak into a host cell during the construction of the host brood and wait for the host to mature, or penetrate inside of host cells after the host matures to the appropriate stage^3^,^4^. In the latter case, females are likely to arrive sequentially at hosts and sequentially start to lay eggs, resulting in varying clutch sizes between females.

Second, *Melittobia* females are considered to disperse for a long distance to find suitable hosts^34^. However, because the hosts of *Melittobia* distribute patchily and their generation time is longer than that of *Melittobia, Melittobia* females can also disperse to neighboring hosts existing within the same patch. In fact, in the present case, within the same bamboo tube of a trap, we observed multiple *Melittobia* females laying eggs in a host cocoon next to another host that was parasitized before and already start to disperse. Such short-range dispersal increases the chance of encountering related *Melittobia* females on the same host^4^. This is in contrast to other parasitoid species, such as *Nasonia vitripennis*, in which females disperse over long distances and females in the same patch are generally unrelated^35^. *Nasonia* is one of the model species for sex allocation, for which we have a good understanding of how females shift their offspring sex ratios in accordance with the prediction of LMC theory^26^. This understanding might help to solve the problem of the extremely female-biased sex ratios in *Melittobia* based on their contrasting dispersal patterns. Theoretically, limited dispersal increases relatedness among females on a patch and favors a female-biased sex ratio by avoiding mate competition among related sons and by increasing the number of mates for the related sons. However, limited dispersal also increases competition for resources among related females and disfavors a female-biased sex ratio. The balance between these two opposing effects depends on the details of the population structure and the mode of dispersal^36^,^37^. The microsatellite markers developed in this study can be used for further comprehensive population genetic analysis, specifically to identify the relatedness among females on sharing the same host and to determine the dispersal pattern of females in natural populations.

## Materials and Methods

### Genomic samples and sequencing

Genomic DNA for the sequencing library was extracted from a pool of 20 females from a laboratory stock culture of *M. australica* (S line^29^) that was collected in Otsu, Shiga, Japan in 2000^17^. DNA extraction was done using DNeasy Blood & Tissue Kit (Qiagen, Hilden, Germany) according to the manufacturer instructions. DNA was eluted in 50 μl of AE buffer and RNase I (Life Technologies, Carlsbad, CA, USA) was added at a concentration of 20 μg/ml to digest the RNA. Genomic DNA library was constructed using Ion Xpress Plus Fragment Library Kit (Life Technologies, Carlsbad, CA, USA). After the fragmentation by the restriction enzyme and the adapter ligation, approximately 400 bp sized fragments (without the length of the adapters) were selected by gel excision (E-gel; Invitrogen, Carlsbad, CA, USA). To determine the appropriate dilution for the template preparation, the library was evaluated and quantified using a Bioanalyser 2100 (Agilent Technologies, Santa Clara, CA, USA). The diluted library was amplified by emulsion PCR using an Ion One Touch 2 (Life Technologies, Carlsbad, CA, USA) and enriched using an Ion One Touch ES (Life Technologies, Carlsbad, CA, USA) with an Ion PGM Hi-Q OT2 Kit (Life Technologies, Carlsbad, CA, USA). Sequencing was performed on an Ion Torrent PGM sequencer with an Ion PGM Hi-Q Sequencing Kit and Ion 318 Chip Kit v2 (Life Technologies, Carlsbad, CA, USA).

### Microsatellite mining and polymorphism check

Reads were scanned for the presence of microsatellite using msatcommander 1.08^38^. Duplicate reads were removed and reads shorter than 150 bp were excluded to facilitate subsequent primer design. Microsatellites with motif lengths of 2 to 6 bp were searched with minimum repeat numbers of 8, 5, 5, 5, and 5 (di−, tri−, tetra−, penta−, and hexanucleotides, respectively). Primers were designed using Primer3 2.2.3^39^. The 5′ ends of forward primers were attached with M13-tails to be annealed by fluorescent-labeled M13 universal primers for a nested PCR below^40^. Selected primer pairs for each locus were tested for amplification and polymorphism in 24 *M. australica* individuals obtained from the field. The field samples were collected between 2011 and 2015 by setting up bamboo traps within a radius of 2 km in and around the campus of Kanagawa University in Hiratsuka, Kanagawa, Japan. The bamboo traps were used for nesting by the leaf cutter bee *Megachile sculpturalis* and its social parasite bee *Chalicodoma sculpturalis* which were parasitized by *Melittobia*. From 12 host prepupae, collected in different traps placed at different spots, one male and one female were sampled per prepupae. This sampling scheme avoids that the individuals of the same sex are relatives and share the same alleles due to identical by descent. In addition, using the primers developed for the Hiratsuka population, we also analyzed samples from the island population of the Yaeyama district, Okinawa, Japan. Yaeyama is about 1,900 km away from the Hiratsuka population and separated by the Pacific ocean. Samples for the analysis were collected from parasitized hosts, which nested in bamboo traps placed in Ishigaki or Iriomote islands between 2009 and 2014. Similar to the Hiratsuka population, one male and one female were sampled from 9 individual hosts collected from different traps to avoid sampling related individuals.

The DNA of *Melittobia* wasps was extracted from their whole bodies by the boiling method described elsewhere^29^ with 120 μl of the squishing buffer (10 mM Tris-HCl [pH 8.2], 1 mM EDTA, 25 mM NaCl). PCR amplification of microsatellite loci was done following the M13-tails technique procedure^40^. The nested PCR reaction mix in a 5 μl volume containing 0.05 μl forward and 0.2 μl reverse primers (10 μM each), 0.2 μl M13 primer (10 μM) labeled a fluorescent dye (6-FAM, VIC, NED or PET), 0.5 μl 10× PCR buffer containing 20 mM MgCl_2_, 0.4 μl dNTPs (2.5 mM each), 0.05 μl Ex Taq (TaKaRa, Shiga, Japan) and 1 μl of the genomic DNA was amplified using an ABI 2720 thermal cycler (Life Technologies, Carlsbad, CA, USA). The PCR temperature profile consisted of 5 min at 94 °C, then 30 cycles of 30 s at 94 °C, 45 s at 60 °C and 45 s at 72 °C, followed by 8 cycles of 30 s at 94 °C, 45 s at 53 °C and 45 s at 72 °C, and a final extension 10 min at 72 °C. The amplified fragments were analyzed using the Peak Scanner software version1.0 (Life Technologies, Carlsbad, CA, USA) after electrophoresing by an ABI 3130 capillary sequencer (Life Technologies, Carlsbad, CA, USA).

### Population genetic analysis and estimation of sib-mating frequency

Population genetic statistics were calculated using GENEPOP 4.2^41^. Due to the haplodiploid sex determination in Hymenoptera species, we tested for linkage disequilibrium (LD) between loci for male (haploid) and female (diploid) individuals separately. Likewise, the observed and expected heterozygosity (*H*_O_ and *H*_E_), the inbreeding coefficient (*F*) and the deficiency of Hardy-Weinberg equilibrium (HWE) were calculated for female individuals only. For the tests of LD and the deficiency of HWE, significance thresholds in multiple comparisons were corrected using the false discovery rate (FDR)^42^. To evaluate the level of genetic isolation between the Hiratsuka and Yaeyama populations, the fixation index (*F*_*ST*_) was calculated. The frequency of sib-mating *α* was estimated from the inbreeding coefficient *F* in a haplodiploid inheritance: *α* = 4 *F*/(3 *F* + 1)^43^. Assuming that all females on a host lay equally sized clutches and the females are unrelated, the number of females laying eggs on a host *n* is given by *n* = 1/*α*, hence *n* = (3*F* + 1)/4*F*^44^, where *n* is the harmonic mean number of the females ovipositing on a host^45^.

## Additional Information

**How to cite this article**: Abe, J. and Pannebakker, B. A. Development of microsatellite markers and estimation of inbreeding frequency in the parasitoid wasp *Melittobia. Sci. Rep.* **7**, 39879; doi: 10.1038/srep39879 (2017).

**Publisher's note:** Springer Nature remains neutral with regard to jurisdictional claims in published maps and institutional affiliations.

## Supplementary Material

Supplementary Table 1

Supplementary Table 2

Supplementary Table 3

## Figures and Tables

**Table 1:** Number and frequency of microsatellite loci identified in reads over 150 bp in *Melittobia australica*.

Motif type	Number	Frequency
Dinucleotide	82750	86.83%
Trinucleotide	7749	8.13%
Tetranucleotide	3723	3.91%
Pentanucleotide	772	0.81%
Hexanucleotide	311	0.33%
Total	95305	100.00%

**Table 2:** Characterization of 20 microsatellite loci for *Melittobia australica* in the Hiratsuka population.

Loci	Accession no.^a^	Repeat motif^b^	Primer sequence (5′-3′)	Size range (bp)^c^	*N*_a_^c^	*H*_O_^d^	*H*_E_^d^	*F*^d^	*P* of HWE^e^
Mau14	LT598812	(AG)^24^	F: CCTATCAGCTTTGTTGGCCG	228–244	3	0.333	0.507	0.353	N.S.
			R: GACAGCTCAACACGCCTAC						
Mau15	LT598824	(AG)^22^	F: TTATGTTGCGGGTGAGTTGC	363–375	3	0.417	0.663	0.382	N.S.
			R: CTGAGCATCGCAATCTGACG						
Mau16	LT598820	(AG)^20^	F: GAAGGTCGATTGGGCAAGTC	275–289	3	0.083	0.627	0.872	<0.001
			R: ATCACGTAGAGTCAGCGCAG						
Mau17	LT598831	(AG)^19^	F: CGCGCAGAACCCTCTAATG	304–312	4	0.250	0.471	0.480	N.S
			R: TACCAGTCTTAGCCACACCC						
Mau19	LT598817	(AG)^17^	F: CTGCGTTGCTCTGAGTTCTG	225–233	3	0.167	0.681	0.763	<0.001
			R: CGTTGTCCGCTCCTTTCAAG						
Mau29	LT598830	(AC)^19^	F: GCAGAGCCTCAAGTTCCAAC	109–127	3	0.333	0.670	0.514	N.S.
			R: CCGAATGCCAGTGTCAGATG						
Mau37	LT598819	(GCT)^18^	F: CCTCTCCCTGTAGTTTCGAAC	153–165	4	0.083	0.554	0.855	<0.01
			R: AACGACGAGCAAGCAAGAAG						
Mau39	LT598823	(TGC)^16^	F: TCGTCGTCCCACTTGAGAAG	207–210	3	0.333	0.663	0.508	N.S.
			R: GTTCTCACGCCTGCACTAC						
Mau40	LT598829	(AGC)^15^	F: AAGAAGAGCCTCCGAAGCG	370–399	3	0.000	0.304	1.000	<0.01
			R: AGACATTGCCGCATAATCGC						
Mau41	LT598818	(TGC)^14^	F: CATTTGTCAAAGTCGCGCAG	279–288	3	0.417	0.663	0.382	N.S.
			R: GCAGACAAAGGTTCGAGAGC						
Mau42	LT598827	(TGC)^13^	F: GGAGCTGTCGTGTGATGATG	190–202	3	0.333	0.663	0.508	<0.05
			R: ATCCGACTCGTTACCCTTCG						
Mau44	LT598816	(ACT)^14^	F: ATTCTGTACGGGAGAGCGTG	350–362	3	0.167	0.685	0.765	<0.01
			R: TAACGAGAGAGCCCATGTCG						
Mau45	LT598828	(ACT)^13^	F: ACATTCTGTACGGGAGAGCG	249–258	3	0.167	0.685	0.765	<0.01
			R: TTTCGGCTGTGTCGCAATAG						
Mau51	LT598814	(TTC)^17^	F: CTAACGCTCCAATGCCAAGC	360–372	3	0.167	0.554	0.709	<0.01
			R: CTCATCAATGGGACCTTCGG						
Mau57	LT598833	(TTG)^18^	F: CAAAGCCTCCAGATTGCACC	178–193	4	0.167	0.743	0.783	<0.001
			R: TGCTCGTCTAGGCCAAGATC						
Mau58	LT598832	(AAC)^16^	F: ATACACGCGGCAGTTCATTG	212–227	4	0.417	0.663	0.382	N.S.
			R: CTCCGTCGACTTCAGCTCTG						
Mau60	LT598815	(GTT)^14^	F: TGCCATAATAGACCCGCACC	224–230	3	0.417	0.663	0.382	N.S.
			R: AGAGACTGGTGAGCATCGTC						
Mau61	LT598834	(AAC)^13^	F: CTATTGTCACACCCGTTCGC	217–235	3	0.167	0.670	0.760	<0.01
			R: TGGAATCACTGCTCCTCCTG						
Mau63	LT598825	(ACGC)^15^	F: ATTGTGCCGGGAAGATGACC	240–306	4	0.167	0.736	0.781	<0.001
			R: AGGACCTCTCGGCTTTCC						
Mau71	LT598835	(AAGC)^13^	F: AATCGTCTCGTCTTGCTCCC	183–210	4	0.167	0.692	0.768	<0.01
			R: AAGTTCCGCCAATTTCGAGC						

^a^Accession number in the European Nucleotide Archive.

^b^Repeat motifs corresponding to the data in the samples used for the sequencing, superscript values indicate the number of repeats.

^c^Size range and the number of alleles (*N*_a_) obtained from the data of 12 male and 12 female individuals.

^d^Observed and expected heterozygosity (*H*_O_ and *H*_E_, respectively) and inbreeding coefficient (*F*) obtained from the data of 12 female individuals.

^e^*P* value for deviations from Hardy-Weinberg equilibrium after correcting for multiple comparisons using FDR.

**Table 3:** Characterization of 20 microsatellite loci for *Melittobia australica* in the Yaeyama population.

Loci	Size range (bp)^a^	*N*_a_^a^	*H*_O_^b^	*H*_E_^b^	*F*^b^	*P* of HWE^c^	*F*_*ST*_ against Hiratsuka^d^
Mau14	230–236	2	0.000	0.366	1.000	<0.05	0.157
Mau15	365–387	2	0.000	0.366	1.000	<0.05	0.456
Mau16	273–289	2	0.000	0.366	1.000	<0.05	0.356
Mau17	296–302	2	0.000	0.366	1.000	<0.05	0.182
Mau19	221–227	2	0.000	0.366	1.000	<0.05	0.702
Mau29	109–130	2	0.000	0.366	1.000	<0.05	0.426
Mau37	128–162	2	0.000	0.366	1.000	<0.05	0.458
Mau39	186–204	2	0.000	0.366	1.000	<0.05	0.544
Mau40	343–384	2	0.000	0.209	1.000	N.S.	0.562
Mau41	250–276	2	0.000	0.366	1.000	<0.05	0.458
Mau42	170–190	2	0.000	0.366	1.000	<0.05	0.407
Mau44	362–373	2	0.000	0.366	1.000	<0.05	0.447
Mau45	261–273	2	0.000	0.366	1.000	<0.05	0.341
Mau51	360–372	2	0.000	0.366	1.000	<0.05	0.458
Mau57	152–162	2	0.000	0.366	1.000	<0.05	0.375
Mau58	169–258	2	0.000	0.366	1.000	<0.05	0.509
Mau60	214–223	2	0.000	0.366	1.000	<0.05	0.430
Mau61	214–247	2	0.000	0.366	1.000	<0.05	0.441
Mau63	221–298	3	0.000	0.523	1.000	<0.05	0.326
Mau71	210–287	3	0.111	0.386	0.720	<0.05	0.401

^a^Size range and the number of alleles (*N*_a_) obtained from the data of 9 male and 9 female individuals.

^b^Observed and expected heterozygosity (*H*_O_ and *H*_E_, respectively) and inbreeding coefficient (*F*) obtained from the data of 9 female individuals.

^c^*P* value for deviations from Hardy-Weinberg equilibrium after correcting for multiple comparisons using FDR.

^d^Fixation index (*F*_*ST*_) between the Hiratsuka and Yaeyama populations obtained from the data of 12 and 9 female individuals in the Hiratsuka and Yaeyama populations, respectively.
